# Crystal structure of 3,4a,7,7,10a-penta­methyl-3-vinyl­dodeca­hydro-1*H*-benzo[*f*]chromen-9-ol isolated from *Sideritis perfoliata*

**DOI:** 10.1107/S2056989016013864

**Published:** 2016-09-05

**Authors:** Ísmail Çelik, Cem Cüneyt Ersanlı, Rahmi Köseoğlu, Hüseyin Akşit, Ramazan Erenler, Ibrahim Demirtaş, Mehmet Akkurt

**Affiliations:** a Department of Physics, Faculty of Sciences, Cumhuriyet University, 58140 Sivas, Turkey; b Department of Physics, Faculty of Arts and Sciences, Sinop University, 57010 Sinop, Turkey; c Department of Physics, Faculty of Sciences, Erciyes University, 38039 Kayseri, Turkey; d Department of Chemistry, Faculty of Arts and Sciences, Gaziosmanpaşa University, 60240 Tokat, Turkey; e Department of Chemistry, Faculty of Natural Sciences, Çankırı Karatekin University, 18100 Çankırı, Turkey

**Keywords:** crystal structure, cyclo­hexane ring, semi-empirical *PM3* method, HOMO, LUMO

## Abstract

The asymmetric unit of the title compound, C_20_H_34_O_2_, contains two crystallographically independent mol­ecules (1 and 2) with similar conformations. In both mol­ecules, the cyclo­hexane rings adopt a chair conformation, while the oxane rings are also puckered. In the crystal, O—H⋯O hydrogen bonds connect adjacent mol­ecules, forming *C*(6) helical chains located around a 2_1_ screw axis and running along the crystallographic *a* axis. The packing of these chains is governed only by van der Waals inter­actions. Semi-empirical *PM3* quantum chemical calculations are in a satisfactory agreement with the structural results of the X-ray structure analysis. The absolute structure was indeterminate in the present experiment.

## Abstract 2

In the two independent mol­ecules in the asymmetric unit of the title compound, the cyclo­hexane rings adopt a chair conformation, while the oxane rings are also puckered. In the crystal, O—H⋯ O hydrogen bonds connect adjacent mol­ecules, forming a *C*(6) helical chain running along the [100] direction.

## Chemical context   

The *Sideritis* genus belonging to the Lamiaceae family is represented by more than 150 species in the world (Duman 2000 ▸). *Sideritis* species have been reported to have a broad spectrum of biological activities such as anti-inflammatory, anti-oxidant, anti-ulcerogenic, analgesic, anti­microbial, anti­proliferative, anti-HIV and anti­feedant activities (González-Burgos *et al.* 2011 ▸), and they have been consumed as teas, as flavoring agents, for therapeutic purposes, *etc*. In particular, *Sideritis* teas have been used for gastrointestinal disorders such as stomach ache and indigestion, to alleviate common colds, fever, flu and sore throats (Topçu *et al.* 2002 ▸). Phytochemical investigations of the species have revealed the presence of terpenes (Fraga *et al.* 2003 ▸), flavonoids, essential oils and other secondary metabolites (Barberan *et al.* 1985 ▸). As part of our studies in this area, we now describe the isolation and structure of the title compound, (I).
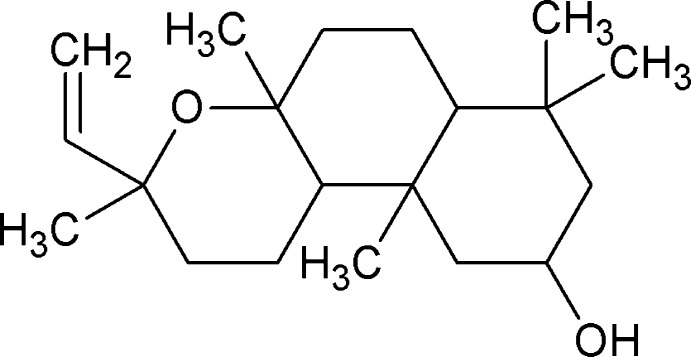


## Structural commentary   

**Figure 1:**
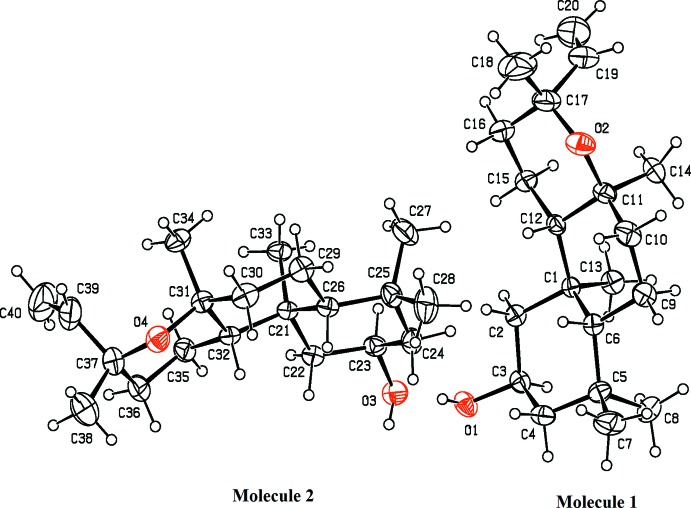
A view of the title compound, showing the atom-numbering scheme. Displacement ellipsoids for non-H atoms are drawn at the 30% probability level. The minor component of the disorder is not shown for clarity.

In the title compound (Fig. 1 ▸), the asymmetric unit contains two crystallographically independent mol­ecules, 1 and 2, with a similar conformations. In mol­ecule 1, the cyclo­hexane ring (C1–C6) attached to the OH group and the central cyclo­hexane ring (C1/C6/C9–C12) each adopt a chair conformation with puckering parameters *Q*_T_ = 0.536 (3) Å, θ = 0.0 (3), φ = 270 (81)° and *Q*_T_ = 0.584 (3) Å, θ = 4.4 (3), φ = 59 (4)°, respectively. The oxane ring (O2/C11/C12/C15–C17) is also puckered, with puckering parameters *Q*_T_ = 0.551 (3) Å, θ = 12.1 (3) and φ = 133.5 (16)°. The equivalent rings in mol­ecule 2 (C21–C16, C21/C26/C29–C32 and O4/C31/C32/C35–C37) have as puckering parameters *Q*_T_ = 0.534 (3) Å, θ = 1.9 (3), φ = 296 (11)°, *Q*_T_ = 0.583 (3) Å, θ = 5.0 (3), φ = 72 (3)° and *Q*_T_ = 0.554 (3) Å, θ = 11.9 (3), φ = 127.2 (15)°, respectively. Bond lengths and angles are within normal range, comparable with each other and with those reported for similar structures in the literature (*e.g.*, Evans *et al.*, 2011 ▸).

## Supra­molecular features   

**Figure 2:**
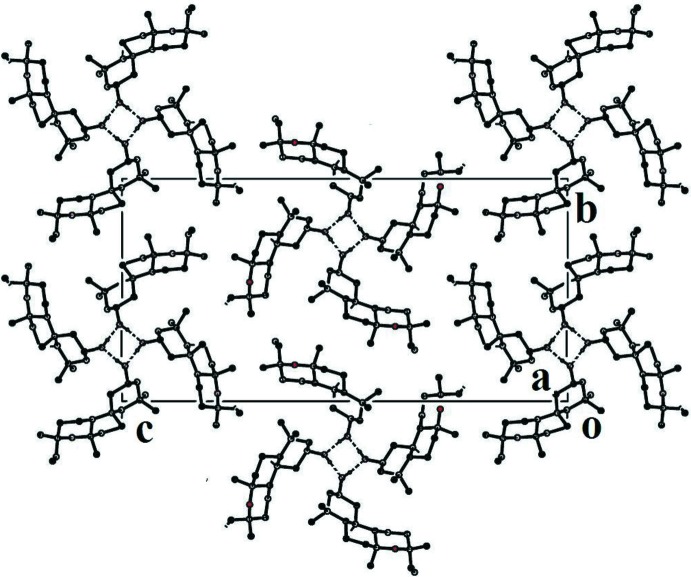
A view along the *a* axis of the crystal packing of the title compound. H atoms not involved in hydrogen bonding (dashed lines) have been omitted for clarity.

Inter­molecular O—H. . . O hydrogen bonds connect adjacent mol­ecules, forming *C*(6) helical chains located around a 2_1_ screw axis running along the crystallographic *a* axis (Table 1 ▸ and Fig. 2 ▸). The crystal packing of these chains is governed only by van der Waals inter­actions. The two asymmetric molecules lead to pseudo-4_1_ symmetry in space group *P*2_1_2_1_2_1_.

## Theoretical calculations   

PM3 (*parameterized model number 3*) is a semi-empirical method for the quantum calculation of the mol­ecular electronic structure in computational chemistry. It is based on the *neglect of differential diatomic overlap* integral approximation. The semi-empirical *PM3* parameterization used in the *MOPAC* program is widely used to derive charges, dipole moments and bond lengths. The computed quantum chemical descriptors include bond lengths, bond angles, torsion angles, atom charges, *HOMO* and *LUMO* energy levels, dipole moment, polarizability, *etc*. In the present case, the geometry of the mol­ecule of the title compound was calculated with a semi-empirical PM3 method (Stewart, 1985 ▸). A spatial view is included in the Supporting information.

The calculated net charges at atoms O1 and O2 are −0.257 and −0.309 e^−^, respectively. The total energy and dipole moment of the title mol­ecule are −3514.7 eV and 1.695 Debye. The *HOMO* and *LUMO* energy levels are −10.36 and 2.71 eV, respectively.

Calculated values for the geometrical parameter are consistent with those obtained by the X-ray structure determination, within the error limits (see Table S1 in the Supporting information), with the sole exception of the angles in the meth­oxy groups. This may be ascribed to the steric inter­actions between adjacent mol­ecules in the crystal structure.

## Synthesis and crystallization   

The aerial part of the plant material (5 g) was extracted with ethyl acetate (3 × 20 mL). After removal of the solvent by rotary evaporator, the extract was subjected to column chromatography (2.5 × 70 cm); sephadex LH-20 (50 g) was used as a stationary phase and methanol was used as a mobile phase with a 0.25 ml min^−1^ flow rate. 16 fractions, each of which was 150 mL, were collected. Similar fractions were combined according to the TLC profile. Further purification was carried out with silica gel column chromatography to isolate the title compound. Colourless prisms were recrystallized from ethanol solution.

## Refinement   

Crystal data, data collection and structure refinement details are summarized in Table 2 ▸. H atoms bound to oxygen were found from difference Fourier maps and their positional parameters were refined with *U*_iso_ fixed at 1.5 times *U*_eq_(O). H atoms bound to carbon were positioned geometrically and allowed to ride on their parent atoms with *U*_iso_ = 1.2*U*_eq_(C) (C—H = 0.93 Å for aromatic, 0.97 Å for methyl­ene and 0.98 Å for methine) and with *U*_iso_ = 1.5*U*_eq_(C) (C—H = 0.96 Å) for methyl H atoms. The absolute structure was indeterminate in the present experiment.

## Supplementary Material

Crystal structure: contains datablock(s) global, I. DOI: 10.1107/S2056989016013864/bg2593sup1.cif

Structure factors: contains datablock(s) I. DOI: 10.1107/S2056989016013864/bg2593Isup2.hkl

Supporting information file. DOI: 10.1107/S2056989016013864/bg2593sup3.pdf

Supporting information file. DOI: 10.1107/S2056989016013864/bg2593sup4.tif

Supporting information file. DOI: 10.1107/S2056989016013864/bg2593Isup5.cml

CCDC reference: 1501445

Additional supporting information: crystallographic information; 3D view; checkCIF report

## Figures and Tables

**Table 1:** Hydrogen-bond geometry (Å, °)

*D*—H⋯*A*	*D*—H	H⋯*A*	*D*⋯*A*	*D*—H⋯*A*
O1—H1*O*⋯O3	0.80 (4)	1.99 (4)	2.784 (3)	170 (4)
O3—H3*O*⋯O1^i^	0.81 (4)	2.00 (4)	2.804 (3)	169 (4)

Symmetry code: (i) 
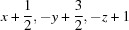
.

**Table 2:** Experimental details

Crystal data	
Chemical formula	C_20_H_34_O_2_
*M*_r_	306.47
Crystal system, space group	Orthorhombic, *P*2_1_2_1_2_1_
Temperature (K)	296
*a*, *b*, *c* (Å)	7.1114 (4), 16.3899 (12), 32.812 (2)
*V* (Å^3^)	3824.4 (4)
*Z*	8
Radiation type	Mo *K*α
μ (mm^−1^)	0.07
Crystal size (mm)	0.14 × 0.11 × 0.08

Data collection	
Diffractometer	Bruker APEXII CCD
Absorption correction	Multi-scan (*SADABS*; Sheldrick, 2003 ▸)
*T*_min_, *T*_max_	0.635, 0.746
No. of measured, independent and observed [*I* > 2σ(*I*)] reflections	36728, 9449, 5384
*R*_int_	0.074
(sin θ/λ)_max_ (Å^−1^)	0.667

Refinement	
*R*[*F* ^2^ > 2σ(*F* ^2^)], *wR*(*F* ^2^), *S*	0.060, 0.130, 1.02
No. of reflections	9449
No. of parameters	413
H-atom treatment	H atoms treated by a mixture of independent and constrained refinement
Δρ_max_, Δρ_min_ (e Å^−3^)	0.17, −0.23
Absolute structure	Flack (1983 ▸), 4144 Friedel pairs
Absolute structure parameter	0.4 (15)

Computer programs: *APEX2* and *SAINT* (Bruker, 2007 ▸), *SHELXS97* (Sheldrick 2008 ▸), *SHELXL2014* (Sheldrick 2015 ▸), *WinGX* (Farrugia, 2012 ▸) and *PLATON* (Spek, 2009 ▸).

## supplementary crystallographic information

### Crystal data

**Table:**

C_20_H_34_O_2_	*F*(000) = 1360
*M**_r_* = 306.47	*D*_x_ = 1.065 Mg m^−^^3^
Orthorhombic, *P*2_1_2_1_2_1_	Mo *K*α radiation, λ = 0.71073 Å
Hall symbol: P 2ac 2ab	Cell parameters from 7578 reflections
*a* = 7.1114 (4) Å	θ = 2.9–25.0°
*b* = 16.3899 (12) Å	µ = 0.07 mm^−^^1^
*c* = 32.812 (2) Å	*T* = 296 K
*V* = 3824.4 (4) Å^3^	Prism, colourless
*Z* = 8	0.14 × 0.11 × 0.08 mm

### Data collection

**Table:**

Bruker APEXII CCD diffractometer	5384 reflections with *I* > 2σ(*I*)
φ and ω scans	*R*_int_ = 0.074
Absorption correction: multi-scan (SADABS; Sheldrick, 2003)	θ_max_ = 28.3°, θ_min_ = 2.9°
*T*_min_ = 0.635, *T*_max_ = 0.746	*h* = −8→9
36728 measured reflections	*k* = −19→21
9449 independent reflections	*l* = −43→42

### Refinement

**Table:**

Refinement on *F*^2^	H atoms treated by a mixture of independent and constrained refinement
Least-squares matrix: full	*w* = 1/[σ^2^(*F*_o_^2^) + (0.0617*P*)^2^ + 0.0101*P*] where *P* = (*F*_o_^2^ + 2*F*_c_^2^)/3
*R*[*F*^2^ > 2σ(*F*^2^)] = 0.060	(Δ/σ)_max_ < 0.001
*wR*(*F*^2^) = 0.130	Δρ_max_ = 0.17 e Å^−^^3^
*S* = 1.02	Δρ_min_ = −0.23 e Å^−^^3^
9449 reflections	Absolute structure: Flack (1983), 4144 Friedel pairs
413 parameters	Absolute structure parameter: 0.4 (15)
0 restraints	

### Special details

**Table:**

Geometry. Bond distances, angles etc. have been calculated using the rounded fractional coordinates. All su's are estimated from the variances of the (full) variance-covariance matrix. The cell esds are taken into account in the estimation of distances, angles and torsion angles
Refinement. Refinement on F^2^ for ALL reflections except those flagged by the user for potential systematic errors. Weighted R-factors wR and all goodnesses of fit S are based on F^2^, conventional R-factors R are based on F, with F set to zero for negative F^2^. The observed criterion of F^2^ > 2sigma(F^2^) is used only for calculating -R-factor-obs etc. and is not relevant to the choice of reflections for refinement. R-factors based on F^2^ are statistically about twice as large as those based on F, and R-factors based on ALL data will be even larger.

### Fractional atomic coordinates and isotropic or equivalent isotropic displacement parameters (Å^2^)

**Table:**

	*x*	*y*	*z*	*U*_iso_*/*U*_eq_	
O1	0.0636 (3)	0.76038 (13)	0.54574 (6)	0.0525 (8)	
O2	0.4700 (3)	0.53531 (14)	0.71395 (6)	0.0656 (8)	
C1	0.2450 (3)	0.70848 (15)	0.65200 (7)	0.0323 (8)	
C2	0.1533 (4)	0.69891 (16)	0.60996 (8)	0.0388 (9)	
C3	0.1457 (4)	0.77677 (17)	0.58487 (8)	0.0400 (9)	
O3	0.3152 (3)	0.65702 (13)	0.50623 (6)	0.0548 (8)	
C4	0.3381 (4)	0.81502 (18)	0.58077 (8)	0.0461 (10)	
O4	0.7389 (3)	0.33641 (12)	0.38715 (6)	0.0558 (7)	
C5	0.4396 (4)	0.83089 (17)	0.62121 (8)	0.0440 (10)	
C6	0.4392 (3)	0.75033 (16)	0.64591 (8)	0.0358 (8)	
C7	0.6443 (5)	0.8531 (2)	0.61049 (11)	0.0723 (12)	
C8	0.3532 (5)	0.90435 (19)	0.64330 (10)	0.0639 (11)	
C9	0.5515 (4)	0.75220 (19)	0.68563 (9)	0.0510 (10)	
C10	0.5874 (4)	0.6660 (2)	0.70119 (9)	0.0576 (11)	
C11	0.4068 (4)	0.61840 (17)	0.70812 (8)	0.0467 (10)	
C12	0.2877 (4)	0.62125 (16)	0.66896 (7)	0.0367 (8)	
C13	0.1074 (4)	0.75550 (18)	0.67990 (8)	0.0460 (10)	
C14	0.3115 (5)	0.6485 (2)	0.74714 (8)	0.0598 (11)	
C15	0.1228 (5)	0.56256 (17)	0.67286 (9)	0.0531 (11)	
C16	0.1983 (6)	0.47707 (19)	0.67885 (10)	0.0679 (14)	
C17	0.3343 (6)	0.4701 (2)	0.71481 (10)	0.0713 (14)	
C18	0.4553 (8)	0.3932 (3)	0.71083 (15)	0.118 (2)	
C19	0.2373 (8)	0.4663 (2)	0.75573 (12)	0.0880 (16)	
C20	0.0645 (9)	0.4471 (3)	0.76331 (15)	0.128 (3)	
C21	0.5044 (3)	0.44774 (15)	0.47669 (7)	0.0333 (8)	
C22	0.4083 (4)	0.53118 (15)	0.47366 (8)	0.0388 (9)	
C23	0.3984 (4)	0.57841 (16)	0.51329 (8)	0.0424 (9)	
C24	0.5903 (4)	0.58643 (18)	0.53327 (9)	0.0519 (10)	
C25	0.6949 (4)	0.50615 (19)	0.53944 (8)	0.0492 (10)	
C26	0.6985 (3)	0.46057 (16)	0.49801 (8)	0.0378 (9)	
C27	0.6092 (6)	0.4573 (2)	0.57489 (9)	0.0747 (15)	
C28	0.8980 (5)	0.5281 (3)	0.55159 (12)	0.0800 (16)	
C29	0.8158 (4)	0.38262 (18)	0.49711 (9)	0.0502 (10)	
C30	0.8551 (4)	0.35654 (18)	0.45324 (9)	0.0514 (10)	
C31	0.6751 (4)	0.34233 (16)	0.42919 (8)	0.0419 (9)	
C32	0.5496 (4)	0.41801 (15)	0.43262 (8)	0.0356 (8)	
C33	0.3720 (4)	0.38850 (17)	0.49895 (8)	0.0453 (10)	
C34	0.5885 (5)	0.26093 (16)	0.44237 (9)	0.0542 (10)	
C35	0.3826 (4)	0.40988 (18)	0.40359 (8)	0.0478 (10)	
C36	0.4571 (5)	0.4026 (2)	0.36037 (9)	0.0619 (11)	
C37	0.6014 (5)	0.3344 (2)	0.35483 (9)	0.0637 (11)	
C38	0.7165 (7)	0.3489 (3)	0.31656 (11)	0.0987 (18)	
C39	0.5169 (7)	0.2505 (2)	0.35154 (12)	0.0843 (16)	
C40	0.3428 (8)	0.2309 (3)	0.34365 (15)	0.122 (2)	
H1O	0.125 (6)	0.727 (2)	0.5337 (12)	0.0980*	
H2A	0.22230	0.65800	0.59470	0.0470*	
H2B	0.02610	0.67880	0.61370	0.0470*	
H3	0.06420	0.81560	0.59910	0.0480*	
H4A	0.32520	0.86640	0.56640	0.0550*	
H4B	0.41600	0.77950	0.56420	0.0550*	
H6	0.50890	0.71210	0.62860	0.0430*	
H7A	0.70930	0.86980	0.63470	0.1080*	
H7B	0.64500	0.89690	0.59110	0.1080*	
H7C	0.70600	0.80640	0.59900	0.1080*	
H8A	0.40310	0.90760	0.67040	0.0960*	
H8B	0.21910	0.89800	0.64460	0.0960*	
H8C	0.38330	0.95340	0.62870	0.0960*	
H9A	0.67060	0.77950	0.68110	0.0610*	
H9B	0.48240	0.78280	0.70600	0.0610*	
H10A	0.66470	0.63700	0.68160	0.0690*	
H10B	0.65680	0.66890	0.72660	0.0690*	
H12	0.36830	0.59640	0.64820	0.0440*	
H13A	0.17640	0.78130	0.70150	0.0690*	
H13B	0.01770	0.71820	0.69130	0.0690*	
H13C	0.04270	0.79630	0.66420	0.0690*	
H14A	0.18570	0.62730	0.74840	0.0900*	
H14B	0.30740	0.70710	0.74710	0.0900*	
H14C	0.38140	0.63000	0.77040	0.0900*	
H15A	0.04510	0.57790	0.69590	0.0640*	
H15B	0.04600	0.56470	0.64840	0.0640*	
H16A	0.26260	0.46010	0.65420	0.0820*	
H16B	0.09370	0.44010	0.68320	0.0820*	
H18A	0.54110	0.39030	0.73340	0.1780*	
H18B	0.52490	0.39530	0.68580	0.1780*	
H18C	0.37570	0.34590	0.71080	0.1780*	
H19	0.31090	0.47940	0.77820	0.1050*	
H20A	−0.01600	0.43330	0.74210	0.1540*	
H20B	0.02070	0.44690	0.79000	0.1540*	
H3O	0.376 (6)	0.680 (2)	0.4887 (11)	0.0840*	
H22A	0.47530	0.56390	0.45380	0.0470*	
H22B	0.28140	0.52330	0.46360	0.0470*	
H23	0.31650	0.54830	0.53200	0.0510*	
H24A	0.57450	0.61250	0.55960	0.0620*	
H24B	0.66760	0.62210	0.51660	0.0620*	
H26	0.76590	0.49780	0.47980	0.0450*	
H27A	0.67140	0.40550	0.57700	0.1120*	
H27B	0.47760	0.44880	0.56990	0.1120*	
H27C	0.62520	0.48710	0.59980	0.1120*	
H28A	0.96390	0.47950	0.55950	0.1200*	
H28B	0.89580	0.56560	0.57400	0.1200*	
H28C	0.96080	0.55290	0.52880	0.1200*	
H29A	0.74900	0.33940	0.51120	0.0600*	
H29B	0.93390	0.39180	0.51120	0.0600*	
H30A	0.92890	0.39840	0.43980	0.0620*	
H30B	0.92870	0.30670	0.45340	0.0620*	
H32	0.62550	0.46210	0.42090	0.0430*	
H33A	0.44450	0.34590	0.51140	0.0680*	
H33B	0.28550	0.36510	0.47980	0.0680*	
H33C	0.30330	0.41750	0.51950	0.0680*	
H34A	0.54640	0.26490	0.47010	0.0820*	
H34B	0.48360	0.24830	0.42510	0.0820*	
H34C	0.68110	0.21860	0.44020	0.0820*	
H35A	0.30950	0.36190	0.41050	0.0570*	
H35B	0.30180	0.45740	0.40590	0.0570*	
H36A	0.51450	0.45400	0.35270	0.0740*	
H36B	0.35230	0.39290	0.34210	0.0740*	
H38A	0.77500	0.40160	0.31800	0.1480*	
H38B	0.63560	0.34670	0.29320	0.1480*	
H38C	0.81150	0.30760	0.31430	0.1480*	
H39	0.59900	0.20710	0.35570	0.1010*	
H40A	0.25380	0.27150	0.33920	0.1460*	
H40B	0.30770	0.17630	0.34240	0.1460*	

### Atomic displacement parameters (Å^2^)

**Table:**

	*U*^11^	*U*^22^	*U*^33^	*U*^12^	*U*^13^	*U*^23^
O1	0.0556 (13)	0.0615 (14)	0.0404 (12)	0.0157 (11)	−0.0096 (10)	0.0025 (10)
O2	0.0720 (15)	0.0624 (15)	0.0625 (14)	0.0127 (13)	−0.0031 (12)	0.0215 (12)
C1	0.0288 (13)	0.0360 (15)	0.0321 (14)	0.0008 (11)	0.0022 (11)	−0.0020 (11)
C2	0.0342 (15)	0.0406 (15)	0.0417 (15)	−0.0004 (12)	−0.0010 (12)	0.0001 (12)
C3	0.0392 (15)	0.0447 (16)	0.0361 (15)	0.0083 (13)	−0.0022 (12)	−0.0010 (12)
O3	0.0585 (14)	0.0481 (13)	0.0578 (13)	0.0106 (11)	0.0178 (11)	−0.0004 (10)
C4	0.0497 (18)	0.0437 (17)	0.0450 (16)	0.0072 (14)	0.0078 (14)	0.0109 (13)
O4	0.0561 (13)	0.0539 (12)	0.0575 (13)	0.0036 (10)	0.0109 (11)	−0.0072 (11)
C5	0.0401 (16)	0.0412 (17)	0.0506 (17)	−0.0040 (13)	0.0038 (13)	0.0035 (13)
C6	0.0280 (13)	0.0409 (15)	0.0385 (14)	0.0009 (12)	0.0045 (12)	−0.0010 (12)
C7	0.054 (2)	0.084 (2)	0.079 (2)	−0.0273 (19)	0.0005 (18)	0.027 (2)
C8	0.079 (2)	0.0426 (18)	0.070 (2)	−0.0066 (17)	−0.0012 (19)	−0.0047 (16)
C9	0.0372 (16)	0.065 (2)	0.0509 (17)	−0.0135 (15)	−0.0063 (14)	0.0059 (15)
C10	0.0403 (17)	0.078 (2)	0.0545 (18)	−0.0012 (17)	−0.0152 (14)	0.0132 (17)
C11	0.0520 (18)	0.0482 (17)	0.0399 (16)	0.0053 (15)	−0.0018 (14)	0.0093 (13)
C12	0.0416 (16)	0.0395 (15)	0.0289 (13)	0.0033 (12)	0.0036 (12)	−0.0004 (11)
C13	0.0369 (15)	0.0509 (18)	0.0501 (17)	0.0036 (14)	0.0101 (13)	−0.0013 (14)
C14	0.076 (2)	0.068 (2)	0.0355 (16)	−0.0062 (18)	−0.0027 (16)	−0.0003 (15)
C15	0.066 (2)	0.0467 (18)	0.0465 (17)	−0.0170 (16)	−0.0032 (16)	0.0043 (14)
C16	0.102 (3)	0.0448 (19)	0.057 (2)	−0.0143 (19)	0.012 (2)	0.0058 (15)
C17	0.103 (3)	0.051 (2)	0.060 (2)	0.008 (2)	0.005 (2)	0.0181 (17)
C18	0.167 (5)	0.063 (3)	0.125 (4)	0.036 (3)	0.021 (4)	0.027 (3)
C19	0.133 (4)	0.065 (2)	0.066 (2)	−0.013 (3)	0.010 (3)	0.022 (2)
C20	0.178 (6)	0.118 (4)	0.089 (3)	−0.063 (4)	0.041 (4)	0.004 (3)
C21	0.0268 (13)	0.0368 (15)	0.0363 (14)	−0.0016 (11)	−0.0019 (11)	0.0078 (12)
C22	0.0325 (14)	0.0433 (16)	0.0407 (15)	0.0019 (13)	−0.0002 (12)	0.0056 (12)
C23	0.0424 (16)	0.0436 (17)	0.0413 (15)	0.0005 (14)	0.0107 (13)	0.0045 (12)
C24	0.0532 (18)	0.0572 (19)	0.0453 (17)	−0.0051 (16)	0.0053 (15)	−0.0095 (14)
C25	0.0435 (17)	0.062 (2)	0.0420 (17)	−0.0046 (15)	−0.0106 (15)	−0.0011 (14)
C26	0.0284 (14)	0.0431 (16)	0.0418 (15)	−0.0043 (12)	−0.0025 (12)	0.0077 (12)
C27	0.096 (3)	0.088 (3)	0.0400 (18)	0.006 (2)	−0.0099 (19)	0.0103 (18)
C28	0.062 (2)	0.094 (3)	0.084 (3)	0.003 (2)	−0.029 (2)	−0.026 (2)
C29	0.0309 (15)	0.0564 (19)	0.0633 (19)	0.0026 (13)	−0.0155 (15)	0.0046 (16)
C30	0.0325 (15)	0.0507 (18)	0.071 (2)	0.0104 (13)	0.0004 (15)	0.0047 (15)
C31	0.0389 (15)	0.0390 (16)	0.0478 (16)	0.0028 (13)	0.0039 (13)	0.0008 (13)
C32	0.0338 (14)	0.0329 (14)	0.0402 (14)	−0.0008 (12)	−0.0008 (12)	0.0067 (12)
C33	0.0374 (16)	0.0485 (17)	0.0501 (17)	−0.0068 (13)	0.0023 (13)	0.0112 (14)
C34	0.0558 (18)	0.0378 (16)	0.069 (2)	0.0038 (15)	−0.0042 (16)	0.0059 (14)
C35	0.0494 (18)	0.0487 (17)	0.0452 (16)	0.0060 (14)	−0.0115 (14)	0.0012 (13)
C36	0.078 (2)	0.064 (2)	0.0438 (18)	0.0015 (19)	−0.0134 (16)	−0.0016 (15)
C37	0.081 (2)	0.063 (2)	0.0471 (18)	−0.002 (2)	0.0030 (18)	−0.0111 (16)
C38	0.128 (4)	0.110 (3)	0.058 (2)	0.000 (3)	0.029 (2)	−0.012 (2)
C39	0.110 (3)	0.070 (3)	0.073 (2)	−0.001 (2)	−0.013 (2)	−0.023 (2)
C40	0.137 (4)	0.100 (4)	0.129 (4)	−0.030 (3)	−0.050 (4)	−0.018 (3)

### Geometric parameters (Å, º)

**Table:**

O1—C3	1.436 (3)	C18—H18A	0.9600
O2—C11	1.447 (4)	C19—H19	0.9300
O2—C17	1.440 (4)	C20—H20A	0.9300
O1—H1O	0.80 (4)	C20—H20B	0.9300
C1—C6	1.555 (3)	C21—C26	1.562 (3)
C1—C12	1.564 (4)	C21—C32	1.559 (3)
C1—C2	1.534 (4)	C21—C22	1.532 (3)
C1—C13	1.546 (4)	C21—C33	1.537 (4)
C2—C3	1.520 (4)	C22—C23	1.515 (4)
C3—C4	1.511 (4)	C23—C24	1.520 (4)
O3—C23	1.437 (3)	C24—C25	1.525 (4)
C4—C5	1.533 (4)	C25—C27	1.538 (4)
O4—C37	1.443 (4)	C25—C28	1.541 (5)
O4—C31	1.455 (3)	C25—C26	1.551 (4)
C5—C7	1.541 (5)	C26—C29	1.526 (4)
C5—C6	1.549 (4)	C29—C30	1.527 (4)
C5—C8	1.534 (4)	C30—C31	1.522 (4)
C6—C9	1.529 (4)	C31—C32	1.532 (4)
C9—C10	1.524 (4)	C31—C34	1.532 (4)
C10—C11	1.520 (4)	C32—C35	1.528 (4)
C11—C12	1.540 (4)	C35—C36	1.519 (4)
C11—C14	1.530 (4)	C36—C37	1.528 (5)
C12—C15	1.522 (4)	C37—C39	1.505 (5)
C15—C16	1.513 (4)	C37—C38	1.518 (5)
C16—C17	1.530 (5)	C39—C40	1.305 (7)
C17—C19	1.511 (6)	C22—H22A	0.9700
C17—C18	1.532 (6)	C22—H22B	0.9700
C19—C20	1.293 (8)	C23—H23	0.9800
C2—H2A	0.9700	C24—H24A	0.9700
C2—H2B	0.9700	C24—H24B	0.9700
O3—H3O	0.81 (4)	C26—H26	0.9800
C3—H3	0.9800	C27—H27A	0.9600
C4—H4B	0.9700	C27—H27B	0.9600
C4—H4A	0.9700	C27—H27C	0.9600
C6—H6	0.9800	C28—H28A	0.9600
C7—H7B	0.9600	C28—H28B	0.9600
C7—H7A	0.9600	C28—H28C	0.9600
C7—H7C	0.9600	C29—H29A	0.9700
C8—H8B	0.9600	C29—H29B	0.9700
C8—H8A	0.9600	C30—H30A	0.9700
C8—H8C	0.9600	C30—H30B	0.9700
C9—H9B	0.9700	C32—H32	0.9800
C9—H9A	0.9700	C33—H33A	0.9600
C10—H10B	0.9700	C33—H33B	0.9600
C10—H10A	0.9700	C33—H33C	0.9600
C12—H12	0.9800	C34—H34A	0.9600
C13—H13C	0.9600	C34—H34B	0.9600
C13—H13A	0.9600	C34—H34C	0.9600
C13—H13B	0.9600	C35—H35A	0.9700
C14—H14C	0.9600	C35—H35B	0.9700
C14—H14A	0.9600	C36—H36A	0.9700
C14—H14B	0.9600	C36—H36B	0.9700
C15—H15A	0.9700	C38—H38A	0.9600
C15—H15B	0.9700	C38—H38B	0.9600
C16—H16A	0.9700	C38—H38C	0.9600
C16—H16B	0.9700	C39—H39	0.9300
C18—H18C	0.9600	C40—H40A	0.9300
C18—H18B	0.9600	C40—H40B	0.9300
			
C11—O2—C17	119.5 (2)	H20A—C20—H20B	120.00
C3—O1—H1O	110 (3)	C19—C20—H20A	120.00
C2—C1—C6	107.9 (2)	C22—C21—C26	107.7 (2)
C2—C1—C12	108.0 (2)	C22—C21—C32	108.10 (19)
C6—C1—C12	106.05 (19)	C26—C21—C32	105.98 (19)
C6—C1—C13	114.7 (2)	C26—C21—C33	114.4 (2)
C12—C1—C13	111.6 (2)	C32—C21—C33	111.7 (2)
C2—C1—C13	108.3 (2)	C22—C21—C33	108.8 (2)
C1—C2—C3	114.6 (2)	C21—C22—C23	114.9 (2)
O1—C3—C2	110.0 (2)	O3—C23—C22	109.8 (2)
O1—C3—C4	111.5 (2)	O3—C23—C24	111.2 (2)
C2—C3—C4	111.4 (2)	C22—C23—C24	111.9 (2)
C3—C4—C5	114.8 (2)	C23—C24—C25	114.9 (2)
C31—O4—C37	119.1 (2)	C24—C25—C27	110.9 (2)
C4—C5—C7	106.7 (2)	C24—C25—C28	106.9 (3)
C4—C5—C8	110.7 (2)	C26—C25—C27	114.7 (2)
C6—C5—C8	114.9 (2)	C26—C25—C28	108.9 (2)
C7—C5—C8	107.5 (2)	C27—C25—C28	107.3 (3)
C4—C5—C6	107.9 (2)	C24—C25—C26	107.9 (2)
C6—C5—C7	108.8 (2)	C21—C26—C25	116.3 (2)
C1—C6—C5	116.4 (2)	C25—C26—C29	115.4 (2)
C5—C6—C9	115.3 (2)	C21—C26—C29	111.2 (2)
C1—C6—C9	111.3 (2)	C26—C29—C30	110.6 (2)
C6—C9—C10	110.8 (2)	C29—C30—C31	112.2 (2)
C9—C10—C11	112.6 (2)	O4—C31—C32	107.8 (2)
O2—C11—C12	108.1 (2)	O4—C31—C34	109.6 (2)
O2—C11—C14	109.3 (2)	O4—C31—C30	103.9 (2)
C10—C11—C14	109.5 (2)	C30—C31—C34	109.0 (2)
C12—C11—C14	116.4 (2)	C32—C31—C34	116.8 (2)
O2—C11—C10	103.9 (2)	C30—C31—C32	109.2 (2)
C10—C11—C12	108.9 (2)	C21—C32—C31	116.2 (2)
C1—C12—C11	115.6 (2)	C21—C32—C35	116.4 (2)
C1—C12—C15	117.3 (2)	C31—C32—C35	109.7 (2)
C11—C12—C15	109.5 (2)	C32—C35—C36	108.5 (2)
C12—C15—C16	108.8 (3)	C35—C36—C37	113.8 (3)
C15—C16—C17	113.2 (3)	O4—C37—C38	103.8 (3)
O2—C17—C19	110.7 (3)	O4—C37—C39	110.1 (3)
C16—C17—C18	110.5 (3)	C36—C37—C38	110.2 (3)
O2—C17—C18	103.5 (3)	C36—C37—C39	114.1 (3)
C18—C17—C19	107.4 (3)	C38—C37—C39	107.4 (3)
C16—C17—C19	113.6 (4)	O4—C37—C36	110.5 (2)
O2—C17—C16	110.7 (3)	C37—C39—C40	128.2 (4)
C17—C19—C20	128.0 (4)	C21—C22—H22A	109.00
C1—C2—H2A	109.00	C21—C22—H22B	109.00
C3—C2—H2A	109.00	C23—C22—H22A	108.00
C3—C2—H2B	109.00	C23—C22—H22B	108.00
C1—C2—H2B	108.00	H22A—C22—H22B	108.00
H2A—C2—H2B	108.00	O3—C23—H23	108.00
C2—C3—H3	108.00	C22—C23—H23	108.00
C4—C3—H3	108.00	C24—C23—H23	108.00
O1—C3—H3	108.00	C23—C24—H24A	109.00
C23—O3—H3O	108 (3)	C23—C24—H24B	109.00
C3—C4—H4B	109.00	C25—C24—H24A	109.00
C5—C4—H4A	109.00	C25—C24—H24B	109.00
H4A—C4—H4B	108.00	H24A—C24—H24B	108.00
C5—C4—H4B	109.00	C21—C26—H26	104.00
C3—C4—H4A	109.00	C25—C26—H26	104.00
C9—C6—H6	104.00	C29—C26—H26	104.00
C1—C6—H6	104.00	C25—C27—H27A	109.00
C5—C6—H6	104.00	C25—C27—H27B	109.00
C5—C7—H7B	109.00	C25—C27—H27C	109.00
C5—C7—H7A	109.00	H27A—C27—H27B	109.00
H7A—C7—H7C	109.00	H27A—C27—H27C	110.00
C5—C7—H7C	109.00	H27B—C27—H27C	110.00
H7A—C7—H7B	109.00	C25—C28—H28A	110.00
H7B—C7—H7C	110.00	C25—C28—H28B	109.00
C5—C8—H8B	109.00	C25—C28—H28C	109.00
C5—C8—H8C	109.00	H28A—C28—H28B	109.00
H8A—C8—H8C	109.00	H28A—C28—H28C	110.00
H8B—C8—H8C	110.00	H28B—C28—H28C	109.00
H8A—C8—H8B	109.00	C26—C29—H29A	110.00
C5—C8—H8A	109.00	C26—C29—H29B	110.00
C6—C9—H9B	109.00	C30—C29—H29A	110.00
C10—C9—H9B	109.00	C30—C29—H29B	110.00
H9A—C9—H9B	108.00	H29A—C29—H29B	108.00
C6—C9—H9A	110.00	C29—C30—H30A	109.00
C10—C9—H9A	109.00	C29—C30—H30B	109.00
C11—C10—H10A	109.00	C31—C30—H30A	109.00
C9—C10—H10A	109.00	C31—C30—H30B	109.00
C9—C10—H10B	109.00	H30A—C30—H30B	108.00
H10A—C10—H10B	108.00	C21—C32—H32	104.00
C11—C10—H10B	109.00	C31—C32—H32	104.00
C11—C12—H12	104.00	C35—C32—H32	104.00
C1—C12—H12	104.00	C21—C33—H33A	109.00
C15—C12—H12	104.00	C21—C33—H33B	110.00
C1—C13—H13A	109.00	C21—C33—H33C	109.00
H13A—C13—H13B	109.00	H33A—C33—H33B	109.00
C1—C13—H13B	110.00	H33A—C33—H33C	110.00
H13B—C13—H13C	110.00	H33B—C33—H33C	109.00
C1—C13—H13C	109.00	C31—C34—H34A	110.00
H13A—C13—H13C	109.00	C31—C34—H34B	109.00
C11—C14—H14B	110.00	C31—C34—H34C	109.00
C11—C14—H14A	109.00	H34A—C34—H34B	109.00
H14A—C14—H14C	109.00	H34A—C34—H34C	109.00
C11—C14—H14C	110.00	H34B—C34—H34C	110.00
H14A—C14—H14B	109.00	C32—C35—H35A	110.00
H14B—C14—H14C	109.00	C32—C35—H35B	110.00
C12—C15—H15B	110.00	C36—C35—H35A	110.00
C16—C15—H15A	110.00	C36—C35—H35B	110.00
H15A—C15—H15B	108.00	H35A—C35—H35B	108.00
C16—C15—H15B	110.00	C35—C36—H36A	109.00
C12—C15—H15A	110.00	C35—C36—H36B	109.00
C15—C16—H16A	109.00	C37—C36—H36A	109.00
C17—C16—H16A	109.00	C37—C36—H36B	109.00
C17—C16—H16B	109.00	H36A—C36—H36B	108.00
H16A—C16—H16B	108.00	C37—C38—H38A	110.00
C15—C16—H16B	109.00	C37—C38—H38B	109.00
C17—C18—H18C	109.00	C37—C38—H38C	109.00
C17—C18—H18A	109.00	H38A—C38—H38B	109.00
C17—C18—H18B	109.00	H38A—C38—H38C	109.00
H18B—C18—H18C	109.00	H38B—C38—H38C	110.00
H18A—C18—H18B	110.00	C37—C39—H39	116.00
H18A—C18—H18C	110.00	C40—C39—H39	116.00
C20—C19—H19	116.00	C39—C40—H40A	120.00
C17—C19—H19	116.00	C39—C40—H40B	120.00
C19—C20—H20B	120.00	H40A—C40—H40B	120.00
			
C17—O2—C11—C10	−169.9 (2)	C15—C16—C17—C19	78.7 (4)
C17—O2—C11—C12	−54.3 (3)	C15—C16—C17—C18	−160.6 (3)
C17—O2—C11—C14	73.2 (3)	C15—C16—C17—O2	−46.6 (4)
C11—O2—C17—C19	−78.7 (4)	C18—C17—C19—C20	−102.8 (5)
C11—O2—C17—C16	48.2 (4)	O2—C17—C19—C20	145.0 (4)
C11—O2—C17—C18	166.6 (3)	C16—C17—C19—C20	19.7 (5)
C12—C1—C2—C3	−165.9 (2)	C32—C21—C22—C23	−165.4 (2)
C13—C1—C6—C5	−68.4 (3)	C33—C21—C26—C25	−68.0 (3)
C13—C1—C2—C3	73.1 (3)	C33—C21—C22—C23	73.2 (3)
C6—C1—C2—C3	−51.7 (3)	C26—C21—C22—C23	−51.3 (3)
C2—C1—C12—C15	−57.2 (3)	C22—C21—C32—C35	−58.3 (3)
C13—C1—C6—C9	66.6 (3)	C33—C21—C26—C29	66.8 (3)
C2—C1—C12—C11	171.2 (2)	C22—C21—C32—C31	170.2 (2)
C12—C1—C6—C9	−57.1 (3)	C32—C21—C26—C29	−56.7 (3)
C13—C1—C12—C15	61.8 (3)	C33—C21—C32—C35	61.3 (3)
C6—C1—C12—C11	55.8 (3)	C26—C21—C32—C31	55.0 (3)
C6—C1—C12—C15	−172.6 (2)	C26—C21—C32—C35	−173.5 (2)
C13—C1—C12—C11	−69.8 (3)	C33—C21—C32—C31	−70.2 (3)
C12—C1—C6—C5	168.0 (2)	C32—C21—C26—C25	168.5 (2)
C2—C1—C6—C9	−172.6 (2)	C22—C21—C26—C29	−172.2 (2)
C2—C1—C6—C5	52.5 (3)	C22—C21—C26—C25	53.0 (3)
C1—C2—C3—C4	53.9 (3)	C21—C22—C23—C24	52.8 (3)
C1—C2—C3—O1	178.0 (2)	C21—C22—C23—O3	176.9 (2)
O1—C3—C4—C5	−177.3 (2)	O3—C23—C24—C25	−176.2 (2)
C2—C3—C4—C5	−54.1 (3)	C22—C23—C24—C25	−53.0 (3)
C3—C4—C5—C8	−74.5 (3)	C23—C24—C25—C28	168.7 (3)
C3—C4—C5—C7	168.9 (2)	C23—C24—C25—C27	−74.6 (3)
C3—C4—C5—C6	52.1 (3)	C23—C24—C25—C26	51.8 (3)
C37—O4—C31—C32	−55.5 (3)	C27—C25—C26—C21	70.9 (3)
C37—O4—C31—C30	−171.2 (2)	C27—C25—C26—C29	−62.1 (3)
C37—O4—C31—C34	72.5 (3)	C24—C25—C26—C29	173.8 (2)
C31—O4—C37—C38	166.7 (3)	C28—C25—C26—C29	58.2 (3)
C31—O4—C37—C39	−78.5 (3)	C28—C25—C26—C21	−168.9 (3)
C31—O4—C37—C36	48.5 (3)	C24—C25—C26—C21	−53.3 (3)
C7—C5—C6—C1	−167.9 (2)	C25—C26—C29—C30	−164.0 (2)
C7—C5—C6—C9	58.9 (3)	C21—C26—C29—C30	60.8 (3)
C4—C5—C6—C9	174.3 (2)	C26—C29—C30—C31	−58.3 (3)
C8—C5—C6—C1	71.5 (3)	C29—C30—C31—C32	53.0 (3)
C8—C5—C6—C9	−61.6 (3)	C29—C30—C31—C34	−75.5 (3)
C4—C5—C6—C1	−52.5 (3)	C29—C30—C31—O4	167.8 (2)
C5—C6—C9—C10	−164.3 (2)	O4—C31—C32—C35	59.4 (3)
C1—C6—C9—C10	60.3 (3)	C34—C31—C32—C21	70.2 (3)
C6—C9—C10—C11	−57.8 (3)	C30—C31—C32—C21	−53.9 (3)
C9—C10—C11—C14	−75.3 (3)	C30—C31—C32—C35	171.6 (2)
C9—C10—C11—C12	53.1 (3)	O4—C31—C32—C21	−166.0 (2)
C9—C10—C11—O2	168.1 (2)	C34—C31—C32—C35	−64.4 (3)
C10—C11—C12—C1	−54.3 (3)	C31—C32—C35—C36	−60.3 (3)
C10—C11—C12—C15	170.6 (2)	C21—C32—C35—C36	165.2 (2)
O2—C11—C12—C15	58.3 (3)	C32—C35—C36—C37	53.9 (3)
C14—C11—C12—C15	−65.0 (3)	C35—C36—C37—O4	−46.2 (4)
C14—C11—C12—C1	70.1 (3)	C35—C36—C37—C38	−160.4 (3)
O2—C11—C12—C1	−166.5 (2)	C35—C36—C37—C39	78.6 (3)
C11—C12—C15—C16	−60.5 (3)	O4—C37—C39—C40	142.0 (4)
C1—C12—C15—C16	165.3 (2)	C36—C37—C39—C40	17.0 (6)
C12—C15—C16—C17	54.8 (4)	C38—C37—C39—C40	−105.6 (5)

### Hydrogen-bond geometry (Å, º)

**Table:**

*D*—H···*A*	*D*—H	H···*A*	*D*···*A*	*D*—H···*A*
O1—H1*O*···O3	0.80 (4)	1.99 (4)	2.784 (3)	170 (4)
O3—H3*O*···O1^i^	0.81 (4)	2.00 (4)	2.804 (3)	169 (4)

Symmetry code: (i) *x*+1/2, −*y*+3/2, −*z*+1.
