# Supplementary material for: Crystal structure of 3,4a,7,7,10a-penta­methyl-3-vinyl­dodeca­hydro-1H-benzo[f]chromen-9-ol isolated from Sideritis perfoliata
Source: Acta Crystallogr E Crystallogr Commun. 2016 Sep 5;72(Pt 10):1380–2. doi: 10.1107/S2056989016013864 (PMC5050758; doi:10.1107/S2056989016013864)
Supplement: Supplementary file 3 [file e-72-01380-sup3.pdf]

Table S1. Comparison of experimental (X-ray) and theoretical (PM3) parameters of the title compound.

Bond (Å)	X-ray (molecule 1)	X-ray (molecule 2)	PM3
O1—C3	1.436 (3)	1.437 (3)	1.4165
O2—C11	1.447 (4)	1.455 (3)	1.4364
O2—C17	1.440 (4)	1.443 (4)	1.4335
C1—C6	1.555 (3)	1.562 (3)	1.5511
C1—C12	1.564 (4)	1.559 (3)	1.5628
C1—C2	1.534 (4)	1.532 (3)	1.5419
C1—C13	1.546 (4)	1.537 (4)	1.5368
C4—C5	1.533 (4)	1.525 (4)	1.5403
C5—C7	1.541 (5)	1.538 (4)	1.5331
C5—C8	1.534 (4)	1.541 (5)	1.5332
C5—C6	1.549 (4)	1.551 (4)	1.5612
C10—C11	1.520 (4)	1.522 (4)	1.5387
C11—C12	1.540 (4)	1.532 (4)	1.5518
C11—C14	1.530 (4)	1.532 (4)	1.5377
C16—C17	1.530 (5)	1.528 (5)	1.5432
C17—C19	1.511 (6)	1.505 (5)	1.5490
C17—C18	1.532 (6)	1.518 (5)	1.5389
Bond Angle (°)			
C11—O2—C17	119.5 (2)	119.1 (2)	119.96
C6—C1—C13	114.7 (2)	114.4 (2)	111.40
C12—C1—C13	111.6 (2)	111.7 (2)	104.85
C2—C1—C13	108.3 (2)	108.8 (2)	108.98
C1—C2—C3	114.6 (2)	114.9 (2)	112.84
O1—C3—C2	110.0 (2)	109.8 (2)	106.61
O1—C3—C4	111.5 (2)	111.2 (2)	111.90
C2—C3—C4	111.4 (2)	111.9 (2)	111.19

C4—C5—C7	106.7 (2)	110.9 (2)	109.07
C4—C5—C8	110.7 (2)	106.9 (3)	109.03
C6—C5—C7	108.8 (2)	114.7 (2)	110.24
C6—C5—C8	114.9 (2)	108.9 (2)	111.87
C7—C5—C8	107.5 (2)	107.3 (3)	106.23
O2—C11—C14	109.3 (2)	109.6 (2)	109.58
C10—C11—C14	109.5 (2)	109.0 (2)	110.50
C12—C11—C14	116.4 (2)	116.8 (2)	109.8
O2—C17—C18	103.5 (3)	103.8 (3)	103.1
O2—C17—C19	110.7 (3)	110.1 (3)	109.5
C16—C17—C18	110.5 (3)	110.2 (3)	109.8
C16—C17—C19	113.6 (4)	114.1 (3)	111.12
C18—C17—C19	107.4 (3)	107.4 (3)	109.20
C17—C19—C20	128.0 (4)	128.2 (4)	113.12
