# Supplementary figures and images for: Crystal structure of 3,4a,7,7,10a-penta­methyl-3-vinyl­dodeca­hydro-1H-benzo[f]chromen-9-ol isolated from Sideritis perfoliata
Source: Acta Crystallogr E Crystallogr Commun. 2016 Sep 5;72(Pt 10):1380–2. doi: 10.1107/S2056989016013864 (PMC5050758; doi:10.1107/S2056989016013864)

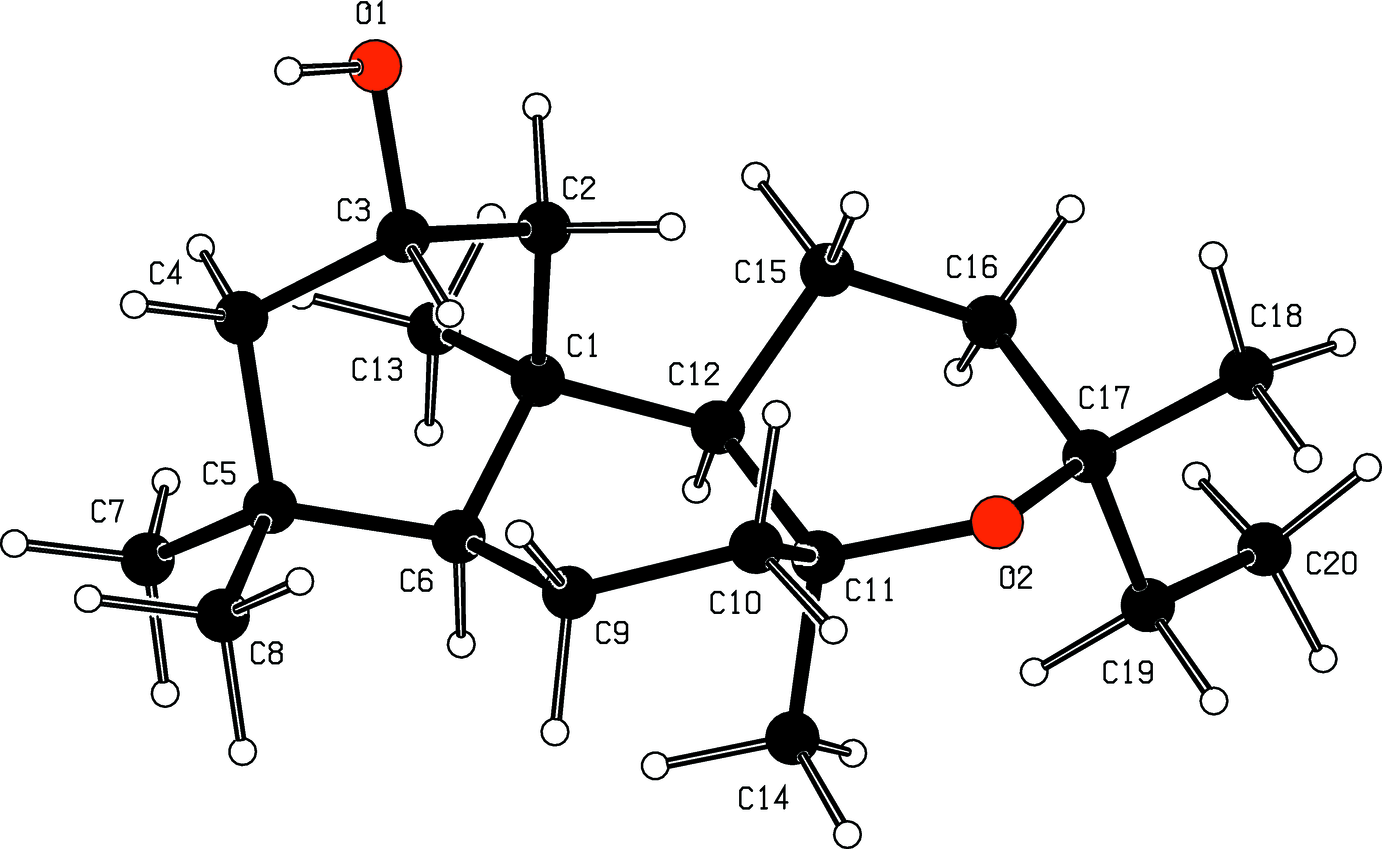

Supplement: Supplementary file 4 [file e-72-01380-sup4.tif]
